# Building toward a standard model for perinatal mental healthcare

**DOI:** 10.3389/fpsyt.2025.1606957

**Published:** 2025-10-03

**Authors:** R. Christopher Sheldrick, Megan Deichen Hansen, Nicole H. Cirino, Mary Kimmel, Courtney King, Deirdre Logan, Rena Menke, Karen Tabb

**Affiliations:** ^1^ UMass Chan Medical School, Shrewsbury, MA, United States; ^2^ Florida State University, Tallahassee, FL, United States; ^3^ Baylor College of Medicine, Houston, TX, United States; ^4^ Department of Psychiatry, Washington University School of Medicine, University of North Carolina System, Chapel Hill, NC, United States; ^5^ Medical University of South Carolina, Charleston, SC, United States; ^6^ University of Michigan, Ann Arbor, MI, United States; ^7^ University of Illinois at Urbana-Champaign, Champaign, IL, United States

**Keywords:** perinatal, mental healthcare, perinatal mental health care model, psychiatry, access programs

## Abstract

**Background:**

Healthcare decision-making relies on models that synthesize complex components such as disease epidemiology, diagnostic accuracy, and treatment efficacy. A healthcare model serves as a framework to integrate health systems research, biological understanding, and diverse perspectives on health, enabling decision-makers to optimize access, quality, cost, and equity. The National Academy of Sciences, Engineering, and Medicine (NASEM) has underscored the need for a shared conceptualization in behavioral sciences to unify definitions and facilitate data synthesis. To achieve this, a standard model of perinatal mental healthcare is imperative.

**Objective:**

We propose the development of a standard model of perinatal mental healthcare, analogous to the Standard Model of Particle Physics, which has guided scientific discovery by defining building blocks, highlighting knowledge gaps, and fostering interdisciplinary collaboration. A standard model for perinatal mental healthcare should function similarly—identifying key components, delineating evidence gaps, and inspiring critical inquiry.

**Methods:**

Our work is informed by our role as an advisory council supporting Perinatal Psychiatry Access Programs, which enhance healthcare systems by providing frontline clinicians with psychiatric consultation, training, and resources. These programs are designed to support evidence-based interventions across screening, therapy, and pharmacotherapy, and have been successfully implemented in multiple states and internationally.

**Conclusion:**

Establishing a robust standard model of perinatal mental healthcare is essential for addressing population-level mental health challenges. Furthermore, collaboration and governance structures for shared resources—akin to Elinor Ostrom’s principles of common-pool resource management—are essential for sustainability. Scientific advances in systems modeling, teamwork, and knowledge-sharing frameworks will be critical to developing an effective, widely accepted model.

## Introduction

Healthcare decisions are often guided by evidence on the natural history and epidemiology of physical diseases, the accuracy of screening and diagnostic tests, and the effectiveness of treatment. For this evidence to be useful, models are needed to weave together these complex components in a simple enough manner to help decision-makers ensure the right balance of access, quality, cost, and equitable use of limited resources. Defined as a “description or representation used to understand the way in which” healthcare works (i.e., its mechanism) (1), a healthcare model can integrate advances in health systems research, understanding of biology, and diverse views on what health means across a broad range of clinical and research settings. In turn, models can inform not only research, but also real-world decisions that require an understanding of how programs work. Drawing on innovations originating in partnerships between research and industry, the National Academy of Sciences, Engineering, and Medicine (NASEM) recently emphasized the need for a shared conceptualization across the behavioral sciences to support “widely shared definitions for key concepts” and to facilitate the ability “to extract and combine data from diverse research contexts and to understand relationships among phenomena.” (2) There is no better way to foster a shared conceptualization than by working toward a standard model of perinatal mental healthcare. We believe that this is the time to work together toward an updated model of perinatal mental healthcare.

Advances in physics offer an example. The Standard Model of Particle Physics synthesizes insights across many areas of study from electricity (e.g., Maxwell’s equations) to gravity (e.g., the general theory of relativity), thereby operationalizing the best, most current theory in the scientific community to describe the most basic building blocks of the universe, and it illustrates how different building blocks can be identified, described, and combined to create a foundation for addressing complex problems. Advances to the Standard Model have resulted in at least 54 Nobel Prizes (3)—not because the Standard Model ever was or ever will be perfect, but because the Standard Model has well-known limits: it doesn’t explain everything, nor does it include everything within its scope. Herein lies one of its strengths: the limits of the Standard Model highlight unsolved problems, evidence gaps, and areas of disagreement. A useful model does not impose uniform solutions; rather, it inspires good questions, thereby helping to engage and direct the contributions of experts of all kinds across nations and decades.

**Figure 1:**
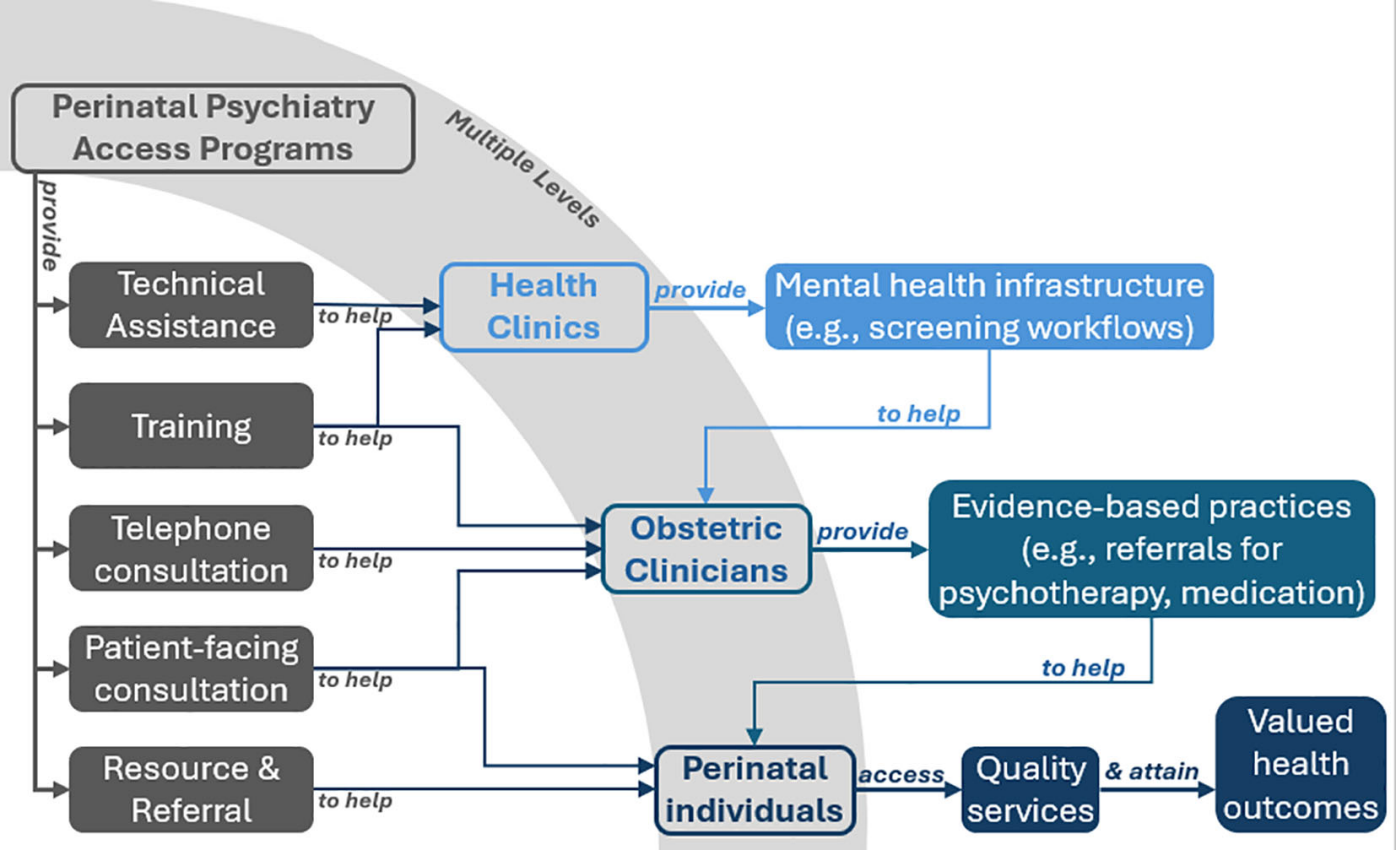
How access programs support perinatal mental healthcare.

As members of an advisory council convened to advance research and evaluation for a systems-level intervention known as Perinatal Psychiatry Access Programs (hereafter referred to as Access Programs), the authors of this paper are developing an applied model to guide our work. To be clear, the science of mental health differs from physics in fundamental ways, and we expect that the most useful models will offer specific and practical applications rather than grand unifying theory. Yet like the Standard Model of Particle Physics, we believe that models can articulate important questions to guide advances in our field. We begin with Access Programs (see **Figure 1**). As described in the USPSTF’s 2023 recommendation on depression screening, Access Programs are “population-based programs that aim to increase access to perinatal mental health care” by building “the capacity of medical professionals to address perinatal mental health and substance use disorders.” As system-level interventions, Access Programs do not function in a silo—they include relationship-building throughout the healthcare system, which can include patient representatives, researchers, clinicians, community stakeholders, payers, and policy makers. Services include training, real-time psychiatric consultation, and resources and referrals—all designed to help front-line providers detect and treat mental health disorders by supporting delivery of a range of evidence-based practices from screening to therapy to pharmacotherapy recommended by the USPSTF and the American College of Obstetrics and Gynecology. First founded in 2012 (4), the Access Program model has scaled rapidly, with similar programs now established in a majority of states (5, 6) and others with national and international scope.

We believe our work to develop and refine a useful model for Access Programs can meaningfully contribute to a standard model of perinatal mental healthcare. A wide range of models are potentially relevant to Access Programs [including determinants and process models of implementation (7), models of health disparities (8, 9), and models of mental health problems themselves (10)]. The question is which are most useful for addressing the problems Access Programs face. Like many developmental phases, the perinatal period represents a dynamic and critical period that touches on all aspects of mental health not only the individual, but also the family and the community. As is true of many interventions, a range of evidence supports the effectiveness of Access Programs. For example, research suggests that psychiatric consultation increases perinatal providers’ knowledge and confidence in treating mental health problems (11), and that providers who use psychiatric consultation services treat more complex cases over time (12). Research also suggests that Access Programs are associated with improved treatment rates and depression outcomes (13) and that they have considerable potential to advance health equity (14–16). Such evidence offers building blocks for a model of mental health care.

Regardless however much evidence there may be for any mental health intervention (Access Programs included), unsolved problems, evidence gaps, or areas of disagreement can persist. A prominent example pertains to mental health screening in the perinatal period (17). In short, the United States Preventive Services Task Force (USPSTF) recommends screening for perinatal mental health problems at scale (18) while the Canadian Task Force on Preventive Health Care (CTF) recommends against screening (19). Despite their difference, the two bodies employ similar methods. Every USPSTF and CTF recommendation is based on a systematic review of the research evidence. In turn, each systematic review is guided by key questions defined in what is known as an “analytic framework,” but could also be described as a hybrid determinant/process model in that it articulates assumptions regarding the process by which interventions influence perinatal mental health outcomes. Despite using similar methods to examine the same evidence, the USPSTF and CTF reach very different conclusions. Thus, it is reasonable to consider whether they are guided by different models. Careful scrutiny reveals that while both analytical frameworks share common tenets (i.e., the need to address critical gaps in perinatal mental health care; the importance of evidence-based care strategies; and the danger of implementing screening in the absence of supportive infrastructure), they articulate different definitions of screening, different standards for what counts as evidence of benefit, and a different burden of proof to achieve a recommendation. In short, the CTF and the USPSTF asked different questions that were grounded in different models. As a result, the CTF and the USPSTF reached different conclusions. Failure to unite on a foundational model resulted in vastly different care recommendations, with significant implications for national perinatal mental health care policy.

Moreover, we suggest that USPSTF and CTF models share limitations that limit progress. To be useful, models must address the most important questions about perinatal mental healthcare. Although both the CTF and the USPSTF recognize the importance of sufficient resources to offer treatment to individuals identified with depression, neither asks key questions or reviews evidence about whether available resources are sufficient. Likewise, neither the USPSTF nor the CTF framework raises key questions about the process of care coordination between screening and better health involving the patient and the provider, the health system in which they meet, and the community in which they live—even though both task forces recognize the importance of these linkages.

Similarly, to be useful, an applied model of any perinatal mental health intervention must allow for and incorporate important key questions as they arise. In the case of Access Programs, what is the best way to address the diversity of implementation when evaluating their common elements? How can we ensure that the benefits of Access Programs outweigh any potential harms, not just for the “average” patient but for all patients? How can we demonstrate that this is the case, ideally reaching standards of evidence that earn recommendations from bodies like the USPSTF and the CTF? Unlike a medication or a form of psychotherapy that is designed to have a direct effect on patients’ symptoms, Access Programs are designed to enhance systems of perinatal mental healthcare—i.e., to address the needs of each individual patient while improving their provider’s knowledge of mental health and its effective treatment. Access Programs focus on human resources by helping to ensure that front-line clinicians have the training and support they need to provide high quality perinatal mental healthcare. As such, evaluation requires a model that recognizes Access Programs as system-level interventions designed to improve the ability of the healthcare system itself to address perinatal mental health by ensuring that resources are sufficient to coordinate care from screening to treatment to symptom remission.

Clearly, developing such a model will require a wide range of technical expertise in quantitative analyses and causal theory (20). But technical advances in modeling will not be enough—we must also find better ways of working together on a standard model as a shared resource by creating a forum that supports principled disagreements to advance and strengthen the field (21, 22). Fortunately, teamwork is increasingly the subject of its own science (23). Rather than imposing hierarchy under a single leader, shared resources like a standard model can be governed in other ways. Elinor Ostrom—the first woman to win the Nobel Prize in economics—dedicated her career to demonstrating that common-pool resources need not end in tragedy; instead, shared governance can promote sustainability, sometimes over centuries (24, 25). Such insights are reflected in the emphasis on team science at the National Institutes of Health (26) and in widescale use of creative commons licenses to share intellectual property as a common pool resource.

As we imagine the future of our field, it is difficult to discern which scientific advances are needed first: in the complexity of systems modeling, in the science of common pool resources, or along some other critical dimension we do not yet recognize. After all, advancing mental health at a population level is not rocket science—it is far more complex and far less predictable (27, 28). To develop a model of perinatal mental health care that is useful not only for Access Programs but also for other multilevel interventions, we strive to engage as many people as possible to make their own unique contributions.
